# Methylsulfonylmethane increases osteogenesis and regulates the mineralization of the matrix by transglutaminase 2 in SHED cells

**DOI:** 10.1371/journal.pone.0225598

**Published:** 2019-12-05

**Authors:** Hanan Aljohani, Linda T. Senbanjo, Meenakshi A. Chellaiah

**Affiliations:** 1 Department of Oncology and Diagnostic Sciences, University of Maryland School of Dentistry, Baltimore, MD, United States of America; 2 Department of Oral Medicine and Diagnostics Sciences, King Saud University School of Dentistry, Riyadh, KSA; Università degli Studi della Campania, ITALY

## Abstract

Methylsulfonylmethane (MSM) is a naturally occurring, sulfate-containing, organic compound. It has been shown to stimulate the differentiation of mesenchymal stem cells into osteoblast-like cells and bone formation. In this study, we investigated whether MSM influences the differentiation of stem cells from human exfoliated deciduous teeth (SHED) into osteoblast-like cells and their osteogenic potential. Here, we report that MSM induced osteogenic differentiation through the expression of osteogenic markers such as osterix, osteopontin, and RUNX2, at both mRNA and protein levels in SHED cells. An increase in the activity of alkaline phosphatase and mineralization confirmed the osteogenic potential of MSM. These MSM-induced effects were observed in cells grown in basal medium but not osteogenic medium. MSM induced transglutaminase-2 (TG2), which may be responsible for the cross-linking of extracellular matrix proteins (collagen or osteopontin), and the mineralization process. Inhibition of TG2 ensued a significant decrease in the differentiation of SHED cells and cross-linking of matrix proteins. A comparison of mineralization with the use of mineralized and demineralized bone particles in the presence of MSM revealed that mineralization is higher with mineralized bone particles than with demineralized bone particles. In conclusion, these results indicated that MSM could promote differentiation and osteogenic potential of SHED cells. This osteogenic property is more in the presence of mineralized bone particles. TG2 is a likely cue in the regulation of differentiation and mineral deposition of SHED cells in response to MSM.

## Introduction

Bone marrow-derived mesenchymal stem cells (BMMSCs) have been found to be an appropriate alternative for cell-based tissue/bone engineering and reconstruction procedures. Embryonic, post-natal, and adult stem cells have been isolated from a variety of tissues and were found to possess vast regenerative potential [1,2]. However, some drawbacks have also been reported, including unpredictable cell behavior, difficulty in manipulation into desired tissue, high risk of rejection and ethical issues [3,4]. Mesenchymal stem cells (MSCs) isolated from oral tissues, such as dental pulp, periodontal ligament, apical papilla, gingival tissue, periosteum, dental follicle, and tooth germ, have been shown to possess demonstrable interactivity with biomaterials used for bone reconstruction [5,6]. Most importantly, dental stem cells possess similar gene expression and comparable regenerative potential to BMMSCs. Advantages of using stem cells from oral tissues are that they can be acquired from a very easily accessible tissue source with a less invasive technique; in addition, a sufficient number of cells can be obtained from the tissue source for any clinical application [7–10].

Previous studies have demonstrated the osteogenic potential of stem cells isolated from the remnant dental pulp of human exfoliated deciduous teeth (SHED cells). These cells displayed a higher proliferative rate and differentiation capacity than adult human dental pulp stem cells *in vitro* [11]. SHED cells represent a population of multipotent stem cells and are pure MSCs. They are not the derivative of hematopoietic cells [8]. SHED cells have unique characteristics compared with bone marrow stromal cells [12]; they have a higher proliferation rate and increased cell population doubling [12,13]. Although SHED cells do not differentiate directly into osteoblasts, they have the potential to induce new bone formation; these cells also exhibit multipotential differentiation. *In vivo* transplantation experiments revealed strong osteogenic capacity [4,11,14,15]. We, therefore, aimed to identify the osteogenic differentiation potential of SHED cells in the presence of methylsulfonylmethane (MSM).

MSM is a sulfur-containing non-toxic natural nutrient found in small quantities in many foods. It is commonly used as a supplement to treat arthritis and other inflammatory conditions [16]. Studies have shown that MSM is an inducer of the differentiation of MSCs into osteoblasts and of osteogenesis. Bone morphogenic proteins (BMPs) have been reported to induce osteogenic differentiation of MSCs [17]. Furthermore, BMP2 in combination with MSM enhanced the mineralization process as compared with cells treated with BMP2 alone *in vitro* [18–20]. MSM was shown to suppress the growth of breast cancer cells by downregulating pathways involving signal transducers and activators of transcription (STAT3 and STAT5b) [21]. However, it was shown to have the opposite effect on the osteogenic differentiation of MSCs via STAT5b activation with mineralization potential [18].

Bone matrix consists of extracellular matrix proteins such as collagen, several non-collagenous proteins, and enzymes, which regulate the process of mineralization [22,23]. Transglutaminase-2 (TG2) is a multifunctional enzyme that has been associated with the matrix maturation and mineralization processes of bone matrix [24,25]. TG2 mediates post-translational modification of both intra- and extracellular proteins by catalyzing the formation of ε-(γ-glutamyl) lysine bonds [26]. Tissue transglutaminase has been reported to be involved with osteopontin (OPN), bone sialoprotein, collagen, and fibronectin, which are the substrates for the formation of bone matrix and the process of mineralization [23].

Since there is limited information regarding the effects MSM on the expression of osteogenic markers and mineralization in SHED cells, we aimed to address these issues *in vitro*. We hypothesized that MSM has the potential to induce the mineralization of SHED cells. TG2 could be an essential target of MSM during the mineralization of SHED cells by promoting cross-linking of matrix proteins of interest (e.g. collagen, OPN, etc.). Here, we report that MSM increases the osteogenic potential of SHED cells by enhancing the levels of osteogenic markers (RUNX2, OPN, and OCN) and hence the mineralization process. TG2 interaction with OPN at day 7 and collagen fibers at day 21 suggest that these proteins may function as scaffolds for the MSM-mediated mineralized matrix formation. These studies provide a foundation for further studies on the role of TG2 on MSM-mediated bone formation by SHED cells.

## Materials and methods

### Reagents

Methylsulfonylmethane (MSM), Ascorbic acid, β-glycerophosphate, Calcein Blue, MTT assay kit, Alkaline phosphatase staining kit, Cystamine, and GAPDH antibody were purchased from Sigma (St. Louis, MO). The following antibodies were bought from the company indicated in parenthesis: Collagen alpha 1 (Col 1; Novus Biological; Littleton, CO); Osteopontin (OPN; Abcam, Cambridge, UK), Transglutaminase (TG2; Abcam, Cambridge, UK), Runt-related transcription factor 2 (RUNX2) and HRP conjugated (mouse or rabbit) secondary antibodies (Santa Cruz Biotechnology, Dallas, TX); Osterix (Millipore, MA, USA). Alizarin Red S (ARS) 2% staining solution was from LifeNet® Cell Technology (CM-0058; Fredrick, MD). Fluorochrome-conjugated secondary antibody Alexa Fluor 488 (#4412) and Alexa Fluor 555 (#8953) (Cell Signaling technology®, Danvers, USA) Super Signal^™^ West Pico Chemiluminescent substrate was bought from Thermo Fisher Scientific (Waltham, Massachusetts**)**. Demineralized and mineralized bone particles were from LifeNet Health^**®**^ **(**Virginia Beach, Virginia).

### Cell culture

SHED cells were a kind gift from Dr. Jacques Nör (University of Michigan, Ann Arbor). Briefly, stem cells were collected from exfoliated deciduous incisors of 7-8-year-old children under approved guidelines set by the National Institutes of Health Office of Human Subjects Research [27]. The pulp was separated from a remnant crown and then digested in a solution of 3 mg/ml collagenase type I (Worthington Biochem, Freehold, NJ) and 4 mg/ml dispase (Roche Molecular Biochemicals) for 1 h at 37°C [28]. Single-cell suspensions were obtained by passing the cells through a 70-μm strainer (Falcon) [29].

Cells were maintained in α-minimal essential medium (MEM) with 10% fetal bovine serum and 1% penicillin/streptomycin. For osteogenic differentiation, osteogenic factors ascorbic acid (50 μM), 5 mM β-glycerophosphate, and 0.05% Gentamicin, were added to the medium, which was then denoted as osteogenic medium (OM). Some cultures were treated with MSM (20 mM) in α-minimal essential medium (denoted as basal medium) with no osteogenic factors. Induction of osteogenic differentiation was conducted at passage 5 or 6.

### Semi-quantitative polymerase chain reaction (RT-PCR) analysis

Cells were seeded at a density of 4 x 10^5^ cells/well in a 6-well plate. Total RNA was extracted from cells incubated with osteogenic medium with or without MSM for 21 days. TRizol reagent was used to extract RNA according to the manufacturer's protocol, and cDNA was synthesized using SuperScript ® III First-strand Synthesis System (Invitrogen, Carlsbad, CA) with 2 μg of total RNA. We used the following steps for PCR reaction with primers shown in Table 1 [30]: 95 ºC for 2 min, 95 ºC for 15 s, 56 ºC for 30 s, 72 ºC for45 s, and 72 ºC for 5 min, for a total of 35 cycles. After amplification, the PCR products were separated by electrophoresis on a 2% agarose gel, stained with GelGreen^™^ and visualized by a G-box [31,32].

**Table 1:** Primers used for semi-quantitative RT-PCR and their respective product size.

Gene	GenBank #	Forward primer	Reverse primer	PCR product size (bp)
**ALP**	NM_014476.5	GCGCAGGACAGGATTAAAGC	TCCACTGCCACAGTCAATCC	246
**OPN**	NM_001040058.1	GAAGTTCTGAGGAAAAGCAGC	GGACTTACTTGGAAGGGTCTCT	161
**OCN**	NM_199173.4	ATGAGAGCCCTCACACTCCT	TGGGGCTCCCAGCCATT	180
**GAPDH**	XM_003273723.2	GCAAATTCCATGGCACCGTC	GGTCCACCACCCTGTTGCTA	391

ALP, Alkaline phosphatase; OPN, Osteopontin; OCN, Osteocalcin

### Immunoprecipitation

Immunoprecipitation was conducted as described previously [33,34]. SHED cells were plated at a density of 4 x 10^5^ cells/well in a 6-well plate and treated with MSM (20 mM) in the presence and absence of TG2 Inhibitor (Cystamine; 2 μM) for 7 and 21 days. Cells cultured in the basal medium were used as controls. Lysates were collected as described above, and protein concentration was measured. An equal amount of protein from each sample was incubated with the antibody of interest overnight at 4°C. A-Sepharose beads were then added to the samples and incubated for 4 hours. The beads were pelleted at 4000 rpm for 5 min and washed three times with ice-cold PBS. The immune complexes were then eluted in electrophoresis sample buffer (62.5 mM Tris-HCl, pH 6.8, 2% SDS, and 10% glycerol) and analyzed on 10% polyacrylamide gels with SDS. For immunoblot analysis, proteins were electrophoretically transferred to a PVDF membrane. Immunoblotting was conducted as described below with antibodies of interest.

### Immunoblotting analysis

Cells were seeded at a density of 4 x 10^5^ cells/well in a 6-well plate. SHED cells were grown in the presence and absence of MSM (20 mM), and cells grown in the osteogenic medium were used as controls. The cells were lysed with 1X radioimmunoprecipitation assay buffer (RIPA) with a protease inhibitor and scraped with a cell scraper. Lysates were placed on ice for 15 minutes and then centrifuged at 15,000 rpm for 15 minutes at 4 ºC. The supernatant was collected, and the protein concentration was determined using the Bradford assay. An equal amount of lysate proteins were analyzed by SDS-PAGE on 10% gel and transferred to PVDF membrane. Membranes were blocked for 2 hours in 5% bovine serum albumin (BSA) in phosphate-buffered saline with tween-20 (PBS-T), ten incubated with the primary antibody of interest in PBS-T at the recommended dilution at 4 ºC overnight. Membranes were washed three times with PBS-T and then incubated with species-specific HRP-conjugated secondary antibody in PBS-T at the recommended dilution at RT for 2h. GAPDH antibody (1:5000 dilution in PBS-T) was used as a loading control. After three washes for 5–10 min each, protein bands were visualized by chemiluminescence using an ECL kit [35,36].

### MTT assay

The MTT colorimetric assay analyzes the number of viable cells by the cleavage of tetrazolium salts added to the culture medium. MSM toxicity was assayed by measuring blue formazan formed from the 3-(4-5-dimethlthiazol-2-yl) 2-5-diphenyl tetrazolium bromide (MTT)) salt by the cleavage of mitochondrial dehydrogenase enzyme (Sigma) as described previously [37]. Cells were seeded at a density of 3000–4000 cells/well in a 96-well flat-bottomed microtiter plate one day before the application of any treatment. Cells were incubated for 21 days at various concentrations of MSM (0, 15, 20, 40 mM). MTT was added to each well and incubated for 4h at 37°C. MTT solubilization solution provided by the manufacturer was added to the wells to stop the chemical reaction and absorbance was read at 570 nm as per instructions provided by the manufacturer (Sigma).

### Alkaline phosphatase (ALP) activity analysis

Two forms of ALP activity assays were used: a visualization staining kit and an enzyme activity assay. Cells were seeded at a density of 4 x 10^5^ cells/well in a 6-well plate containing coverslips and treated with OM for 7 days. ALP staining was performed to detect the level of ALP according to the instructions provided by the manufacturer (Sigma) and as described previously [38]. Briefly, after the removal of medium, cells were washed twice with PBS. They were then fixed with a fixing solution provided in the kit for 30 seconds. Coverslips were rinsed in deionized water and incubated with the alkaline-dye mixture for 15 min in the dark at room temperature. Cells were washed sequentially, first with deionized water, two times (2 min each) and then quickly one time with tap water. Coverslips were air-dried and then mounted on a glass slide with mounting medium Vectashield H-1000 (Vector labs, USA). The slides were viewed using phase-contrast microscopy, and images were captured using a Nikon Eclipse TE 2000- inverted light microscope using 4X objective.

To evaluate the level of the ALP enzyme activity, an ALP colorimetric assay was performed. Cells were seeded at a density of 4 x 10^5^ cells/well in a 6-well plate in the presence or absence of MSM (20 mM) for 0, 7, 14, and 21 days. Cells were washed with cold PBS three times and collected with lysis buffer (50 mM Tris, 0.1% Triton-X100, 1mM MgCl_2_, 100 mM glycine). Lysates were centrifuged at 14,000 rpm for 5 min. An equal amount of supernatant protein was used as triplicates in a 96-well plate to measure the activity. P-Nitrophenyl phosphate (10 μl; Sigma) was added to each well, and the absorbance was measured at 405 nm using a microplate reader (Cytation3 image reader) with software (Gen5 version 2.09).

### Alizarin Red S staining

Cells were seeded at a density of 4 x 10^5^ cells/well in a 6-well plate with and without MSM (20 mM) and incubated for 21 days. Cells grown in osteogenic medium were used as a positive control. To evaluate the effect of MSM on matrix mineralization, SHED cells were washed with PBS three times and fixed with absolute ethanol for 30 min at room temperature. After aspiration of ethanol, 2% Alizarin red stain solution was added to each well until the cells were covered completely, then incubated at room temperature for 45 min in the dark as described previously [38]. Subsequently, wells were washed with deionized water three times to remove unincorporated excess dye. The plates were then scanned with EPSON Perfection V200 Photo scanner. Magnified pictures of the wells were taken using phase-contrast microscopy and images were captured using Nikon Eclipse TE 2000- inverted light microscope using 10X objective.

### Calcein blue staining

SHED cells grown in the presence and absence of MSM (20 mM) were labeled for calcein as described previously [39,40]. Briefly, calcein blue stock solution (3.1 x 10^−3^ M.) made with KOH, was added to the medium 1h before fixation to a final concentration of 30 μM. The cells were washed three times with PBS and fixed with 4% formaldehyde in PBS for 10 min. Calcein-labeled cells were imaged using the Cytation 3 image analyzing system (Gen5 version 2.09).

### Immunofluorescence analysis

SHED cells were plated on chamber-slides at a density of 4 x 10^5^ cells/well and treated with MSM (20 mM) in the presence and absence of TG2 Inhibitor (Cystamine; 2 μM) for 7 and 21 days. Cells cultured only with basal media were used as a negative control. Cells were washed three times with PBS for 5 min each and fixed in 4% paraformaldehyde in PBS for 15 minutes. Cells were then blocked in a blocking buffer containing 10% FBS and 1% Triton X-100 in PBS, for 2 hours. Subsequently, cells were incubated with the primary antibody of interest at the dilutions recommended by the manufacture overnight at 4°C. Cells were washed three times with PBS for 5 min each and incubated with fluorochrome-conjugated secondary antibody for 2 hours in the dark at room temperature. Subsequently, cells were washed three times with PBS for 5 min each. Chambers were removed and cells were added with mounting media and covered with coverslips [36,41,42]. The slides were viewed and photographed with the Cytation3 image analyzing system (Gen5 version 2.09).

### Statistical analysis

Quantitative data are expressed as the mean ± SD and statistical significance was determined using two-way ANOVA or Student T-test when applicable (Graph Pad Inc, San Diego, CA). The level of significance was set at P< 0.05.

## Results

### Osteogenic medium induced the expression of osteogenic markers in SHED cells

**Fig 1:**
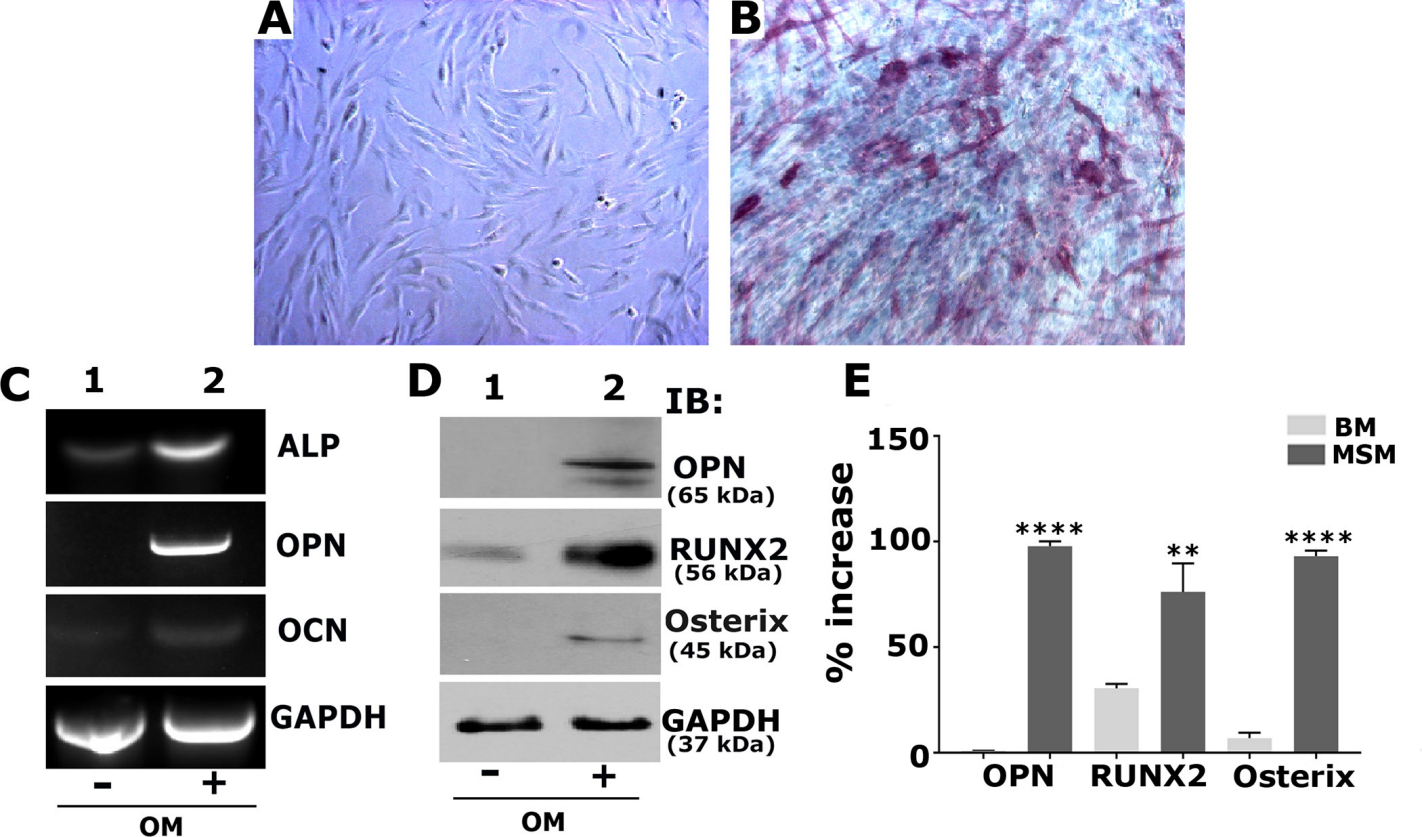
Osteogenic medium (OM) induces the expression of osteogenic markers at mRNA and protein levels in SHED cells. **A and B:** Phase-contrast micrographs of SHED cells at 7 days in the presence of basal (A) or osteogenic medium. Alkaline phosphatase (ALP) stained cells are shown in Panel B. Magnification is 100X. **C-E:** SHED cells treated with basal (-) or osteogenic medium (OM; (+)). RT-PCR (C) and immunoblotting (D) of indicated osteogenic markers to determine mRNA and protein levels, respectively. GAPDH was used as a loading control in C and D. **E.** Expression of OPN, RUNX2, and Osterix proteins were quantitated from three different experiments, and the expression levels are provided as the percent increase for each protein. Error bars represents SD. **** p< 0.001; **p<0.01 vs. basal medium (-).

SHED cells were treated with basal (Fig 1A) or an osteogenic medium (OM; Fig 1B) for 7 days. ALP activity is one of the early markers of osteoblast differentiation. The ability of SHED cells to differentiate into osteoblast-like cells was confirmed by ALP staining (Fig 1B). It is apparent that SHED cells incubated with OM displayed an increase in the number of differentiated cells. Also, these cells formed a multilayered cell-sheet and were positive for ALP staining (Fig 1B). This is indicative of the mineralization potential of differentiating SHED cells. SHED cells incubated with basal medium with no ascorbic acid or β-glycerophosphate (henceforth termed BM) formed spindle-shaped cells with no positive staining for alkaline phosphatase (Fig 1A).

Cells cultured in BM or OM were analyzed for the expression of osteoblast-specific markers of interest at mRNA and protein levels by RT-PCR and immunoblotting analyses, respectively. RUNX2 is an essential transcriptional modulator of osteoblast differentiation, which regulates osteoblast marker genes including Col 1, OPN, and OCN. Therefore, we first determined the levels of these differentiation markers for osteoblasts in SHED cells grown with OM (Fig 1C, lane 2) as compared with cells grown in BM (Fig 1C, lane 1) by RT-PCR analysis. The mRNA expression of ALP, OPN, and OCN increased significantly in response to OM for 7 days. We then determined the expression levels of osteoblast differentiation markers (OPN, RUNX2, and Osterix) at the protein level by immunoblotting analysis. Osterix is another protein indispensable for osteoblast differentiation, and it is a downstream gene of RUNX2. Osterix is required for the expression of osteogenic markers (Col 1, OPN, and OCN) [43,44]. A significant increase in OPN, RUNX2, and Osterix (Fig 1D, lane 2) suggest that SHED cells can be differentiated into osteoblast-like cells that have the potential for osteogenesis in the presence of OM. The levels of these proteins were either very minimal or not observed in cells grown in BM (Fig 1D, lane 1). Expression levels were measured three different experiments; data were compiled and provided as a percent increase in the presence of OM as compared with cells incubated with the BM (Fig 1E).

### MSM induced the differentiation of SHED cells into osteoblast-like cells

#### a. MSM did not affect the viability of SHED cells

**Fig 2:**
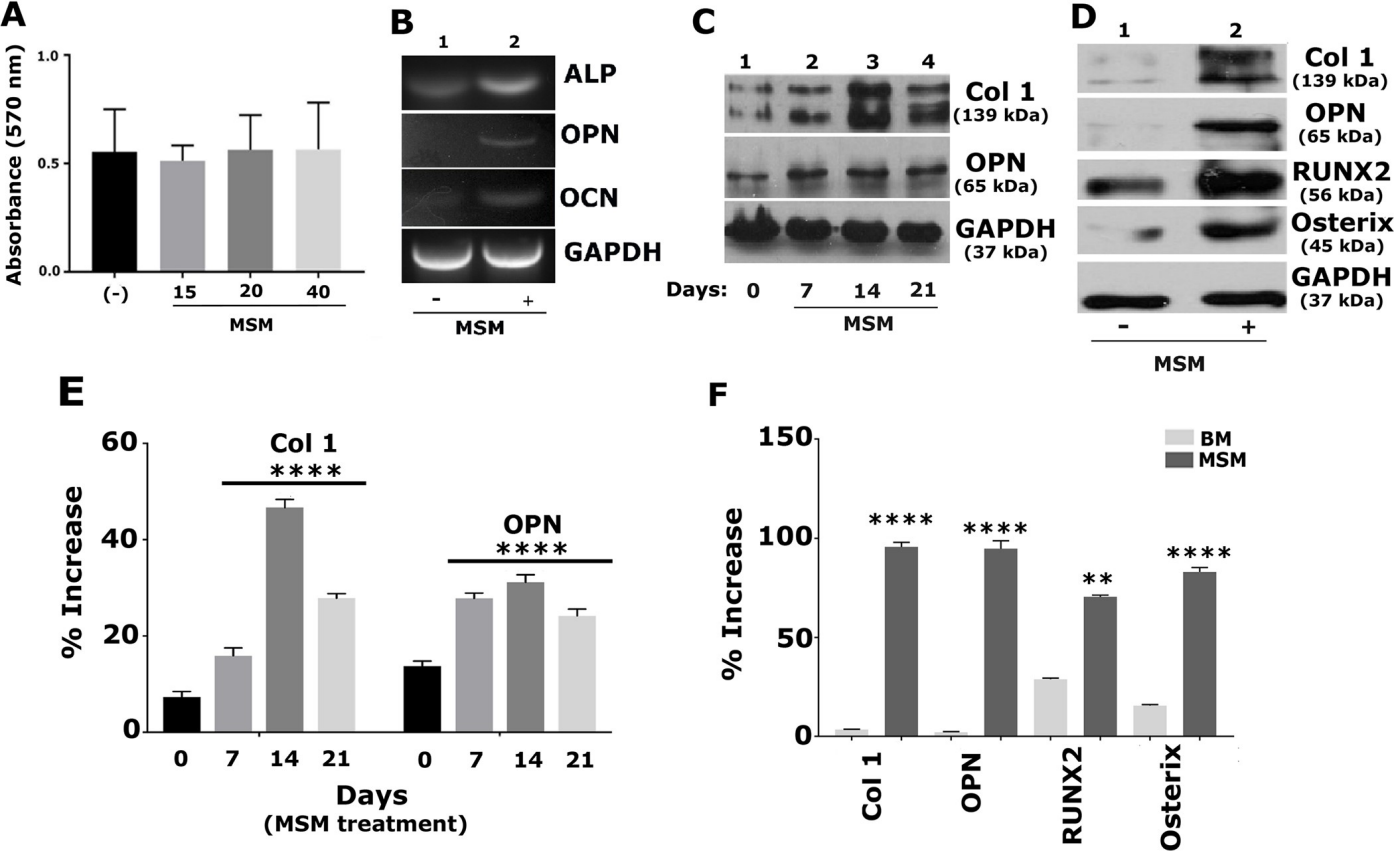
MSM increases the expression of osteogenic markers at mRNA and protein levels. The time-(A) and dose-dependent (C and E) effects of MSM in SHED cells **A.** An MTT assay was conducted in quadruplicate at the indicated concentrations. Error bar represents SD (n = 3). **B.** RT-PCR analysis: The expression levels of osteogenic markers such as ALP, OPN, and OCN were measured in SHED cells treated with MSM (+; 20mM) (B, lane 2). Cells treated with BM alone with no MSM (-) were used as controls (B, lane 1). **C.** Immunoblotting (IB) analysis: Equal amounts of protein lysates (10 μg) from SHED cells treated with MSM for 0, 7, 14, and 21 days (C, lanes 1–4) were used for IB analysis with antibodies to Col 1 and OPN. **D.** IB analyses were conducted with Col 1, OPN, RUNX2, and Osterix antibodies in lysates made from SHED cells treated with MSM for 21 days. BM treated cells (-) were used as a control. The percentage expression is shown from three independent experiments. Error bar represents SD. **** p< 0.001; **p<0.01 vs. BM (-) treated cells. GAPDH was used as a loading control (B-D).

Before characterizing the effects of MSM on the differentiation of SHED cells, we tested the cytotoxicity of MSM using an MTT assay. SHED cells were treated with various concentrations of MSM (0, 15, 20, 40 mM) for 21 days to ensure that long-term exposure to MSM does not affect cell viability. No notable toxicity was observed at varying concentrations of MSM (Fig 2A). Therefore, we chose a 20mM concentration of MSM for all the experiments described below.

#### b. MSM enhances the expression of osteoblast differentiation and osteogenesis markers in SHED cells

First, we analyzed the expression levels of osteoblast-specific markers in SHED cells incubated with BM supplemented with MSM (20 mM) (Fig 2) as we did in cells incubated with OM (Fig 1). The expression levels were measured at mRNA and protein levels by RT-PCR (Fig 2B) and immunoblotting (Fig 2C–2F), respectively. Time-course analysis demonstrated a time-dependent increase in Col 1 protein from days 7 to 21; the increase was greater at days 14 and 21 after treatment with MSM (Fig 2C-top panel; lanes 2–4). However, an increase in OPN was observed at day 7, and this increase remained the same at days 14 and 21 (Fig 2C-middle panel; lanes 2–4). Cells at 0 days demonstrated a basal level expression of OPN and Col 1 (lane 1)

As observed in OM treated cells (Fig 1C–1E), RT-PCR and immunoblotting analyses demonstrated an increase in the expression levels of osteoblast marker genes at mRNA (ALP, OPN, and OCN; Fig 2B) and protein (Col 1, OPN, RUNX2, Osterix; Fig 2D) levels as compared with cells grown in BM with no MSM ((-), lane 1 in Fig 2B and 2D). The percentage of protein expression is shown from three independent experiments (Fig 2E and 2F). These findings support our hypothesis that MSM has the potential to promote the differentiation of SHED cells into osteoblast-like cells, and the effect of MSM is comparable to OM.

### MSM induced the osteogenic potential of SHED cells

**Fig 3:**
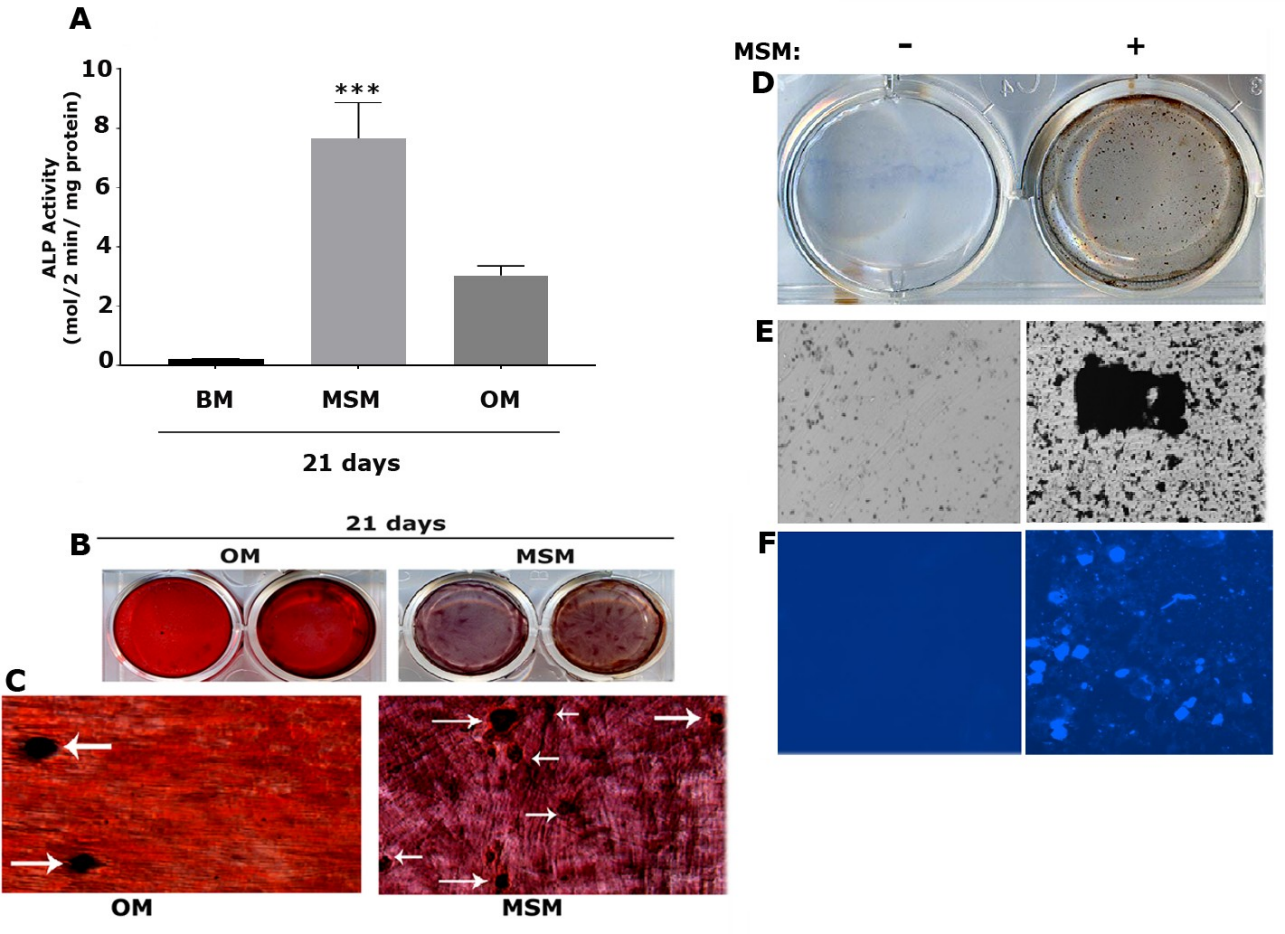
Detection of mineralized matrix and calcium deposits in response to MSM treatment for 21 days. **A.** ALP activity was determined in SHED cells treated with MSM for 21 days. SHED cells treated with osteogenic medium (OM) and basal medium for 21 days were used as controls. Data are expressed as the mean with error bars representing SD (n = 3). ****p<0.0001 vs. OM-treated cells for 21 days. **B and C:** Mineralization was assessed by Alizarin red staining (ARS) in SHED cells treated with MSM for 21 days. Cells treated with osteogenic medium (OM) and MSM are shown in duplicate (B). Representative magnified phase-contrast micrographs (40X magnification) are shown in C. Arrows indicate mineralized nodules in OM and MSM panels (C). Data shown are representative of three independent experiments. **D-F:** Analysis of mineralized nodule formation by Von Kossa (VK; panels D and E) and calcein blue (CB; panel F) staining in vitro. An enlarged view of a VK stained nodule is shown in E (+). Fluorescence analysis of the calcein bound mineral nodules containing calcium are shown in F (+). Magnification is 100X in E and F. Cells in BM (-) was used as a control. Data shown are representative of three independent experiments.

To delineate the osteogenic potential of MSM, we first determined the ALP activity. MSM increased ALP activity in a time-dependent manner. Moreover, MSM induced ALP activity 6–7 fold by day 21 as compared with the effect observed with OM (Fig 3A). Determination of calcium deposits is a good indication of osteogenesis in vitro by MSM. ARS is widely used to detect mineralized nodules *in vitro* in cell cultures [42]. Therefore, ARS staining was conducted in cells treated with MSM or kept in OM for 21 days (Fig 3B). More mineralized nodules were observed in cells treated with MSM than with OM. Besides bigger nodules (indicated by arrows in Fig 3C), several smaller size nodules were also seen in MSM-treated cells. Furthermore, the matrix looked more fibrous and dense with deposits of calcium, which are stained dark in MSM-treated cells at day 21 (Fig 3C). Calcium in the mineralized nodules were also determined using Von Kossa calcium staining and Calcein labeling of SHED cells treated with MSM (+). Cells grown in BM were used as controls. Black deposits represent calcium in the mineralized nodules in Von Kossa calcium stained cells (+, Fig 3D and 3E). Fluorescent (blue) staining of mineralized nodules by calcein labeling (+, Fig 3F) validates the observations shown with ARS and Von Kossa Calcium staining in MSM-treated cells. These results indicate that MSM has clear effects, not only on the differentiation of osteoblast-like cells, but also on the mineralization of the matrix. The bone matrix formed by MSM was very dense and revealed deposits of calcium salts.

### MSM induced the interaction of TG2 with osteopontin and collagen in a time-dependent manner

Many RGD-peptide-containing matrix proteins (OPN, collagen type 1, fibronectin, laminin, and thrombospondin) have been identified as substrates for transglutaminases (TG) [45–48]. Studies have shown that TG2 regulates differentiation and mineralization in the SAOS-2 cell line [49]. However, the role of TG2 in the regulation of differentiation and mineralization of SHED cells in response to MSM treatment has not been studied. An increase in the expression of osteogenic markers and the formation of mineralized matrix prompted us to determine the levels of TG2 in MSM-treated cells. We also used a TG2 inhibitor (Cystamine) to check whether modulating the activity of TG2 has any impact on the expression of osteogenic markers.

#### a. Analysis of the expression levels of TG2 in MSM-treated cells and its interaction with OPN

**Fig 4:**
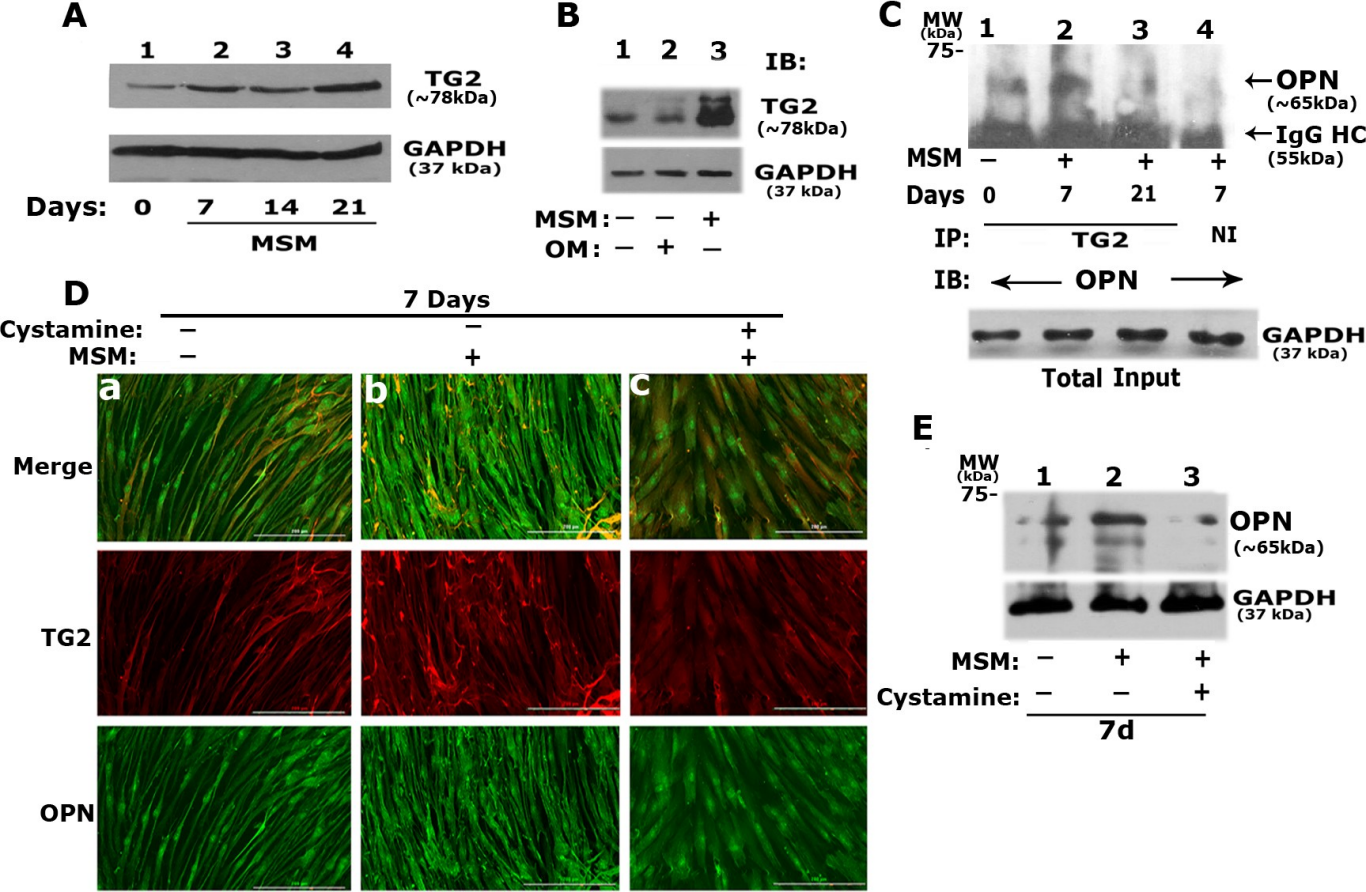
Effect of MSM on the interaction of TG2 with OPN in SHED cells treated with MSM in vitro. **A.** Time-dependent effect of MSM treatment on the total protein level of TG2 was determined by immunoblotting (IB) analysis with an antibody to TG2. SHED cells were treated with MSM for 0, 7, 14, and 21 days (Fig a, lane 2–4). An equal amount of lysate protein (10 μg) was used. **B.** Comparison of TG2 levels in SHED cells incubated with OM (lane 2) or treated with MSM (lane 3) for 21 days. Cells grown in BM (lane 1) were used as controls. IB was conducted with an antibody to TG2. IB with an antibody to GAPDH was conducted after stripping in A and B, and used as a loading control. **C.** Analysis of the interaction of OPN with TG2 was determined by immunoprecipitation and IB analyses. An equal amount of protein lysates (10 μg) were used for immunoprecipitation with a TG2 antibody. Immunoprecipitates were subjected to IB with an antibody to OPN. IB of the total lysates with an antibody to GAPDH indicating the amount of protein from each sample were used for immunoprecipitation/IB analyses shown in panel C. **D.** Immunofluorescent analysis of the effect of TG2 inhibitor on the colocalization of TG2 and OPN in SHED cells treated with MSM (b) and MSM/Cystamine (c) for 7 days, Cells grown in BM (a) were used as controls. Imaging was conducted with the Cytation3 imager. Distribution of OPN and TG2 are shown either together (merge) or separately in green (OPN) and red (TG2) panels. Scale bar, 200μm. **E.** Immunoblotting analysis: The effect of cystamine on the cellular levels of OPN was determined in SHED cells treated with (lane 3) and without cystamine (lane 2) in the presence of MSM. Cells grown in BM (lane 1) were used as controls (-). GAPDH was used as a loading control. Data shown are representative of three independent experiments.

A time-dependent increase in total cellular levels of TG2 was observed from days 7 to 21 (Fig 4), and the increase was maximal on day 21 (Fig 4A lane 4). Also, the expression level of TG2 in MSM-treated cells (Fig 4B, lane 3) was similar to cells grown in OM (Fig 4B, lane 2). TG2 expression was minimal in control cells not treated with MSM or incubated with OM (Fig 4B, lane 1).

Since OPN is one of the substrates for TG2, we proceeded to determine the interaction of TG2 with OPN by immunoprecipitation analysis with an antibody to TG2. Immunoprecipitates were made with TRand 3). Immunoprecipitation with non-immune serum (lane 4) was used as a control. Coprecipitation of OPN with TG2 was observed at day 7 (Fig 4C, lane 2). Although OPN is expressed at 21 days (Fig 2C), its interaction with TG2 is significantly reduced (Fig 4C, lane 3). Furthermore, OPN expression (Fig 2C) and TG2/OPN interactions (Fig 4C, lane 1), were observed to a lesser extent in cells grown in BM. This corresponds with a decrease in the ALP activity or mineralization in these cells (Figs 1 and 3).

#### b. Analysis of the effects of Cystamine on the levels of OPN and TG2/OPN interaction:

To further determine the effect of MSM on TG2 activity, we used cystamine, a well-known inhibitor of TG2. Immunofluorescence analysis demonstrated that SHED cells grown in BM (Fig 4D, panel a) or treated with MSM for 7 days (panel b) were more elongated in shape. Colocalization of TG2 and OPN was minimal (panel a in Fig 4D), consistent with the co-precipitation analysis shown in Fig 4C (lane 1). In addition to elongated shape, cells treated with MSM displayed a fibrillar and diffused staining patterns for both TG2 (red) and OPN (green) in the cytoplasm (panel b in Fig 4D) without any colocalization in most of the cell. However, a characteristic stippled colocalization pattern (Merge in panel b) was observed in these cells. Conforming stippled staining was also observed in the red (TG2) and green (OPN) images (Fig 4B). Neither the fibrillar distribution nor the stippled colocalization of TG2 and OPN was observed in cells treated with cystamine in the presence of MSM (panel c in Fig 4D). These cells were thin, flat, and polygonal in shape. The nuclei of these cells were rich in OPN as compared with the cytoplasm. However, OPN distribution was considerably lower than that seen in cells treated with MSM.

The immunoblotting analysis also showed a significant decrease in OPN protein level in cystamine treated cells (Fig 4E, lane 3) compared with the MSM-treated cells (Fig 4E, lane 2). The OPN level was detected the following order: MSM> BM ((-) MSM) > Cystamine+MSM. An increase in OPN levels and a unique colocalization pattern of TG2/ OPN occurred 7 days following MSM treatment, suggesting that OPN may play an important role in the initiation of the mineralization process.

#### c. Analysis of the effects of Cystamine on the levels of collagen and TG2/collagen interaction

**Fig 5:**
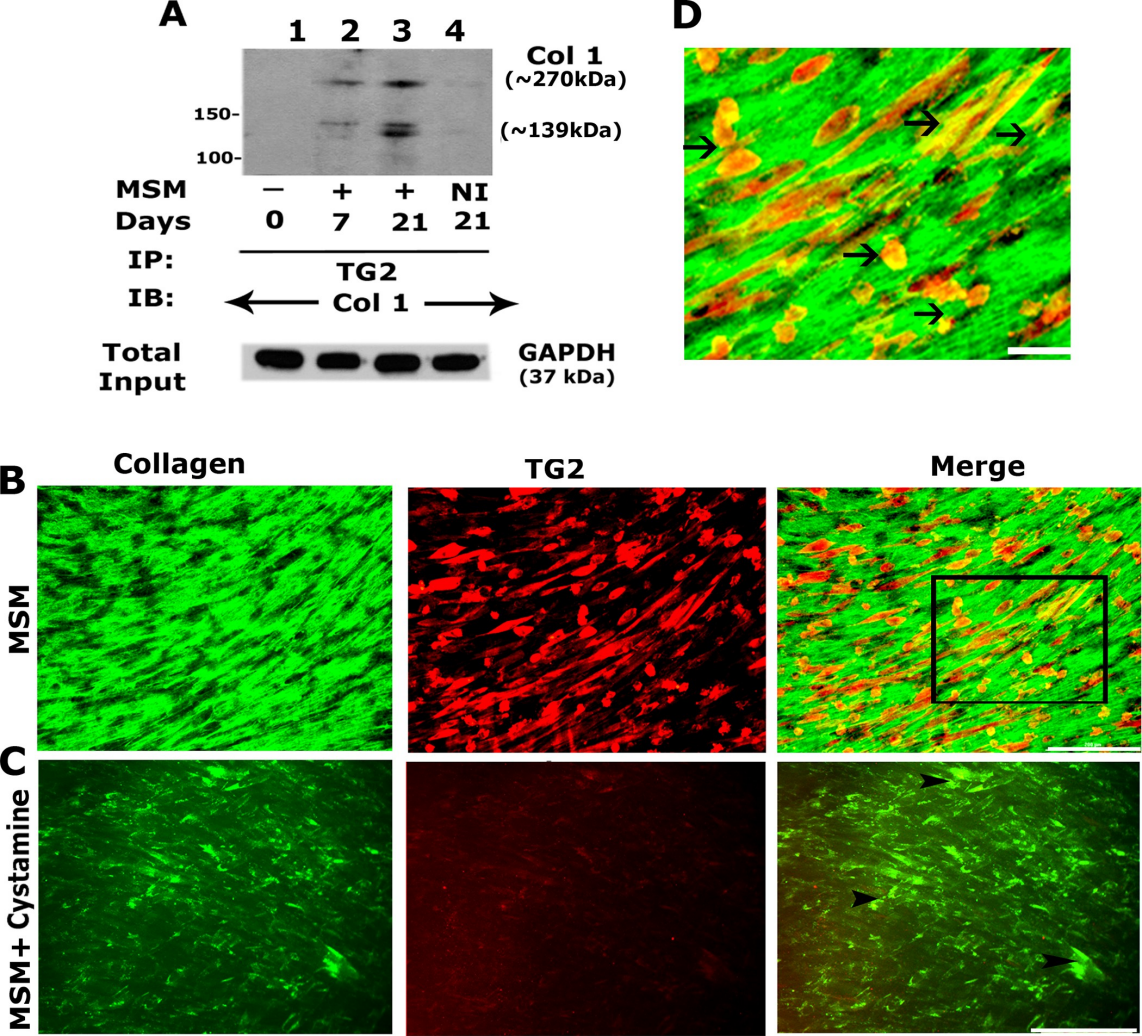
Effect of MSM on TG2 interaction with Collagen I. **A**. Immunoprecipitation and Immunoblotting (IB) analyses. SHED cells were treated with MSM for 0, 7, and 21 days (Fig A, lanes 1–4). An equal amount of lysate protein (10 μg) was immunoprecipitated with an antibody to transglutaminase 2 (TG2) (lanes 1–3) or species-specific non-immune serum (NI, lane 4). Immunoprecipitates were immunoblotted with an antibody to collagen 1 (Col 1). IB of the total lysates with an antibody to GAPDH indicates that an equal amount of protein from each sample was used for immunoprecipitation. **B and C:** Immunofluorescence analysis of the localization of TG2 and collagen in SHED cells. SHED cells were treated with MSM (B) and MSM/cystamine for 21 days (B and C) and immunostained with an antibody to collagen (green) and TG2 (red). Merge panel (left) shows colocalization of TG2 and collagen I. The rectangle in the merge panel of C defines the area of the image which is magnified in panel D. Arrowheads in the merge panel of C and arrows in D point to regions of colocalization (yellow) of collagen 1 and TG2. Scale bar, 200 μm in B and C; 50 μm in D. Data shown are representative of three independent experiments.

TG2 crosslinks extracellular matrix proteins with integrin in primary cells of the human osteoblast lineage grown on collagen/vitronectin-coated supports [50]. A significant increase in collagen at days 14 and 21 (Fig 2C, lanes 3 and 4) prompted us to determine its interaction with TG2 by immunoblotting analysis (Fig 5A). TG2 immunoprecipitates made from MSM treated cells for 21 days demonstrated an increased interaction of collagen with TG2 ((Fig 5A, lane 3) as compared with untreated cells (lane 1) or those treated with MSM for 7 days (lane 2). Immunofluorescent analyses showed cystamine reduced not only the levels of collagen but also TG2 (Fig 5B and 5C). Higher magnification of indicated areas shows Col 1 was more organized into fibrils (Fig 5D) compared to cells treated with TG2 inhibitor, and Col 1 is more dispersed and intercellular (Fig 5C).

### MSM induced mineralization in SHED cells incubated with mineralized bone particles to a greater extent than with demineralized bone particles

**Fig 6:**
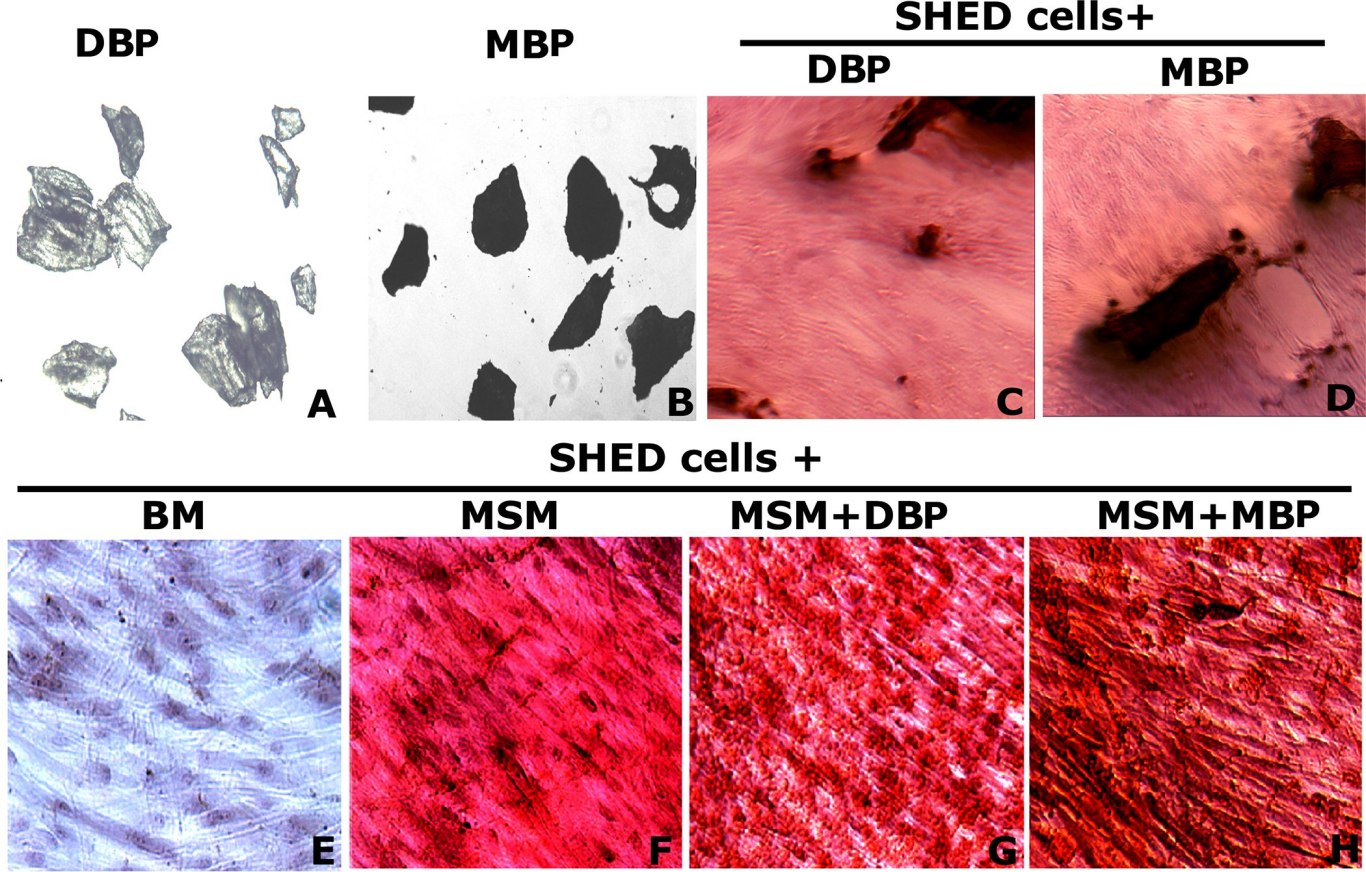
Effect of MSM and bone particles on the mineralization of SHED cells. Phase-contrast micrographs of A and B: the morphology of demineralized (DBP) and mineralized (MBP) bone particles; C and D: SHED cells incubated with DB and MB bone particles with no MSM; E: Cells treated with BM only; F: Cells treated with MSM in BM; G and H: Cells treated with DBPs and MBPs. SHED cells were kept in indicated treatments for 14 days except C and D and then ARS stained (Panels E-H). In C and D, cells were added with indicated BPs and images were taken after 24h. Magnification is X100. Data shown are representative of three independent experiments.

Osteoblasts use the surrounding matrix as a template for mineral deposition [51]. Here we tested the effectiveness of demineralized (DBP) and mineralized (MBP) bone particles on the mineralization process of SHED cells in the presence of MSM for 14 days (Fig 6G and 6H). Cells grown in BM (Fig 6E) and MSM only (Fig 6F) were used as controls. After 14 days in culture, cells were stained with ARS. Pictures are shown in areas with no bone particles to provide a clear view of osteogenic induction (G and H). MB particles in the presence of MSM appeared to have better osteogenic potential than the ones added with DBP/MSM (G) or MSM only (F) *in vitro*. Our initial characterizations with MSM and SHED cells suggest that these are promising candidates for osteoinduction purposes in treating bone defects. In terms of osteoinduction, MBPs appear to have an advantage over DBPs, although detailed analyses on cell proliferation, differentiation, and determination of osteogenic markers are lacking and must be further examined in the future.

## Discussion

In this study, we sought to determine the effect of MSM on the osteogenesis of SHED cells. The use of craniofacial stem cells has been shown to be suitable for oral bone regeneration [14]. Ours is the first study to demonstrate that SHED cells are responsive to MSM in differentiation and mineralization processes. Based on the response of the SHED cells to MSM, we believe that SHED cells may fall in the category of craniofacial stem cells and are therefore promising candidates for tissue engineering. MSM promoted the expression of osteogenic markers at the mRNA level in MSCs in a dose-dependent manner [18]. MSM had no impact on the survival or proliferation of SHED cells when treated with increasing concentrations for 21 days. After evaluating the ability of SHED cells to undergo osteogenesis in the presence of osteogenic medium (OM), we confirmed the effect of MSM on the osteogenesis of SHED cells.

The current study shows that MSM has similar effects as OM on the expression of osteogenic markers at the mRNA (ALP, OPN, and OCN) and protein levels (Col 1, RUNX2, Osterix, and OPN) in SHED cells treated with MSM for 21 days. Our observations suggest that the effect of MSM on SHED cells is equivalent to the effects shown in MSCs with OM [18,19]. RUNX2 is a critical transcriptional factor, and the expression of RUNX2 is associated with osteoblast differentiation [52,53]. An increase in RUNX2 expression suggests that it may have a regulatory role in the expression of its target genes such as OPN and OCN.

MSM has been shown to enhance growth hormone signaling and osteoblast differentiation through the Jak2/STAT5b pathway in osteoblast-like cells (MG-63 and UMR-106) and MSCs [18]. We did not use BMP2 or any growth factor with MSM in our studies. However, our data suggest that MSM induced effects in SHED cells mimic the effects of BMP2 or growth hormone in MSCs and osteoblast-like cells (MG-63 and UMR-106). Although cell lines used were different in these studies (23, 24), our studies are in agreement with these studies that MSM could enhance the osteoblast lineage properties of SHED cells independent of BMP2 or growth hormone.

The ability of the proliferation of SHED cells is reflected in the time-dependent increase in the activity of ALP from days 7 to 21. ALP activity represents an early cell differentiation marker for osteoblasts [54] rather than with stemness [55]. SHED cells demonstrated a time-dependent increase in ALP activity from days 7 to 21. However, a considerable decrease in ALP activity was observed with OM. Other studies have shown that after the initial peak, the ALP level decreases, and the expression of collagen occurs, onto which the mineral deposition occurs [56]. Also, the high expression of OPN and OCN was observed at the final stage from days 14 to 28 [57,58]. Our observations with SHED cells and MSM treatment is different from others in the expression of osteogenic markers and ALP activity. Expression of OPN was higher at 7 days and declined at days 14 and 21; expression of collagen type 1 was high at days 14 and 21 in SHED cells treated with MSM. The differences in the expression pattern of osteogenic markers may be dependent on the cell type and culture conditions. These initial characterizations suggest that SHED cells may have unique characteristics as suggested by others. One possible reason for the differences in ALP activity and the expression of osteogenic markers mentioned above may be due to the higher proliferation rate and increased cell population doubling of SHED cells [12,13]. A limitation here is the lack of elucidation of the signaling mechanisms associated with the differentiation and mineralization processes. Future studies will determine the mechanism by which MSM regulates the differentiation and expression of osteogenic markers in SHED cells *in vitro*.

Mineralization is the final stage of osteogenesis. In our study, we demonstrated the ability of MSM to induce ECM deposition and nodule formation by SHED cells. These analyses showed that the mineralization process is either equal or more in MSM treated cells as compared to OM. As shown by others with human dental pulp stem cells [59], we showed here that SHED cells are able to differentiate into osteoblast-like cells and expresses osteogenic markers in response to MSM. Another interesting finding is the time-dependent increase in the levels of TG2 in MSM-treated cells. Consistent with the observations by others [60], increased TG2 levels correspond with the increased levels of ECM proteins (OPN and collagen type 1) and mineralization. The interaction of TG2 with OPN may have a role in the differentiation and initiation of mineralization, which is followed up by collagen through its interaction with TG2. In our studies with SHED cells, TG2 crosslinking activity was shown to either partially or fully provide support for the differentiation process. Cystamine, a TG2 inhibitor, significantly reduced nodule formation. Cells cultured in basal medium and treated with MSM/cystamine demonstrated nuclear existence of TG2. As shown by others in rabbit liver cells [61,62], cystamine- treated cells demonstrated a cell morphology similar to stem cells. These cells lack cell-to-cell adhesion and a significant decrease in the levels of OPN and collagen, as well as the differentiation of SHED cells into osteoblast-like cells. Results obtained in cystamine-treated cells confirmed the possible requirement of TG2 in the differentiation and mineralization processes of SHED cells in vitro.

The other critical element of regenerative medicine is the use of scaffolds, which provide structural support for the regenerative material and block endothelium migration to the site of surgery [63,64]. As a preliminary study, we looked at the effect of bone particles (MB and DB) on mineralization in the presence of SHED cells and MSM. Previous studies by others demonstrated that mineralized freeze-dried mineralized bone allografts had more significant bone formation potential than demineralized bone allografts [65]. Therefore, we used both types of bone particles in the presence of MSM and SHED cells. We have shown here an increase in mineralization with MB particles as compared with DB particles in vitro. A limitation in this study is the lack of *in vivo* studies to validate the choice of the materials and their effects on the remodeling process. This is our next focus.

## Conclusions

In summary, we have shown here the effect of MSM on the osteogenic differentiation mineralization potential of SHED cells. An increase in osteogenic factors such as OPN and collagen 1 corresponds with an increase in nodule formation and mineralization. An increase in nodule formation and mineralization was related to the levels and activity of TG2 in SHED cells. TG2 likely plays a role in the differentiation and cross-linking of matrix proteins during the mineralization process. Also, we have shown here that mineralization is higher with MB particles than with DM particles. Accordingly, our study suggests that MSM, SHED cells, and mineralized bone particles are promising components for bone regeneration or osteoinduction processes in treating bone defects.

## Supporting information

S1 Fig Osteogenic medium (OM) induces the expression of osteogenic markers at mRNA and protein levels in SHED cells. Uncropped raw data provided for 1C (RT-PCR) and 1D (immunoblotting analysis).
(TIF)

S2 Fig MSM increases the expression of osteogenic markers at mRNA and protein levels. Uncropped raw data provided for 2B (RT-PCR) as well as 2C and D (immunoblotting analysis).
(TIF)

S3 Fig Effect of MSM on the interaction of TG2 with OPN in SHED cells treated with MSM in vitro. Uncropped raw data provided for 4A-C and E (immunoblotting analysis).
(TIF)

S4 Fig Effect of MSM on TG2 interaction with Collagen I. Uncropped raw data provided for 5A (immunoblotting analysis). (TIF)

## Abbreviations

BMMSCs: Bone marrow mesenchymal stem cells
SHED: Stem cells from human exfoliated deciduous teeth
MSM: methylsulfonylmethane
MSCs: mesenchymal stem cells
ROS: reactive oxygen species
Col 1: Collagen alpha 1
OPN: Osteopontin
RUNX2: Runt-related transcription factor 2
OM: Osteogenic Medium
ARS: Alizarin Red S
ALP: Alkaline Phosphatase
KOH: Potassium Hydroxide
PBS: Phosphatase buffered saline
MTT: 3-(4-5-dimethlthiazol-2-yl) 2-5-diphenyl tetrazolium bromide salt
FBS: Fetal Bovine Serum
α-MEM: α-minimal essential medium
