# Supplementary figures and images for: Methylsulfonylmethane increases osteogenesis and regulates the mineralization of the matrix by transglutaminase 2 in SHED cells
Source: PLoS One. 2019 Dec 5;14(12):e0225598. doi: 10.1371/journal.pone.0225598 (PMC6894810; doi:10.1371/journal.pone.0225598)

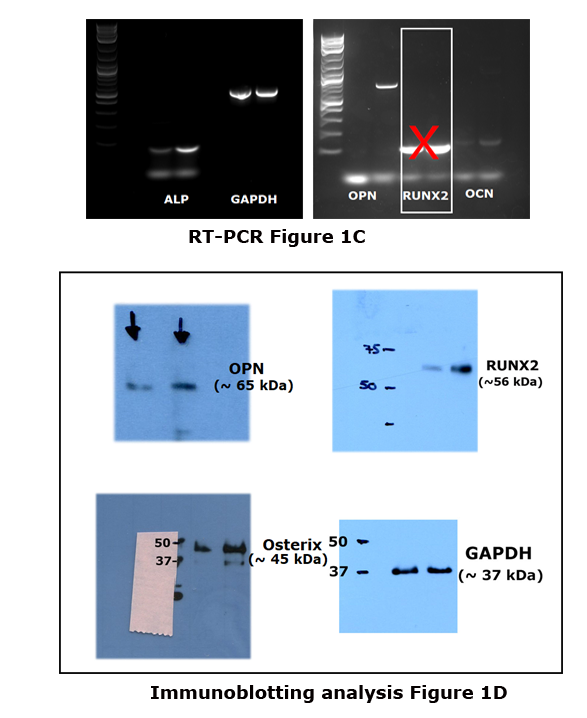

Supplement: S1 Fig — Uncropped raw data provided for 1C (RT-PCR) and 1D (immunoblotting analysis). (TIF) [file pone.0225598.s001.tif]

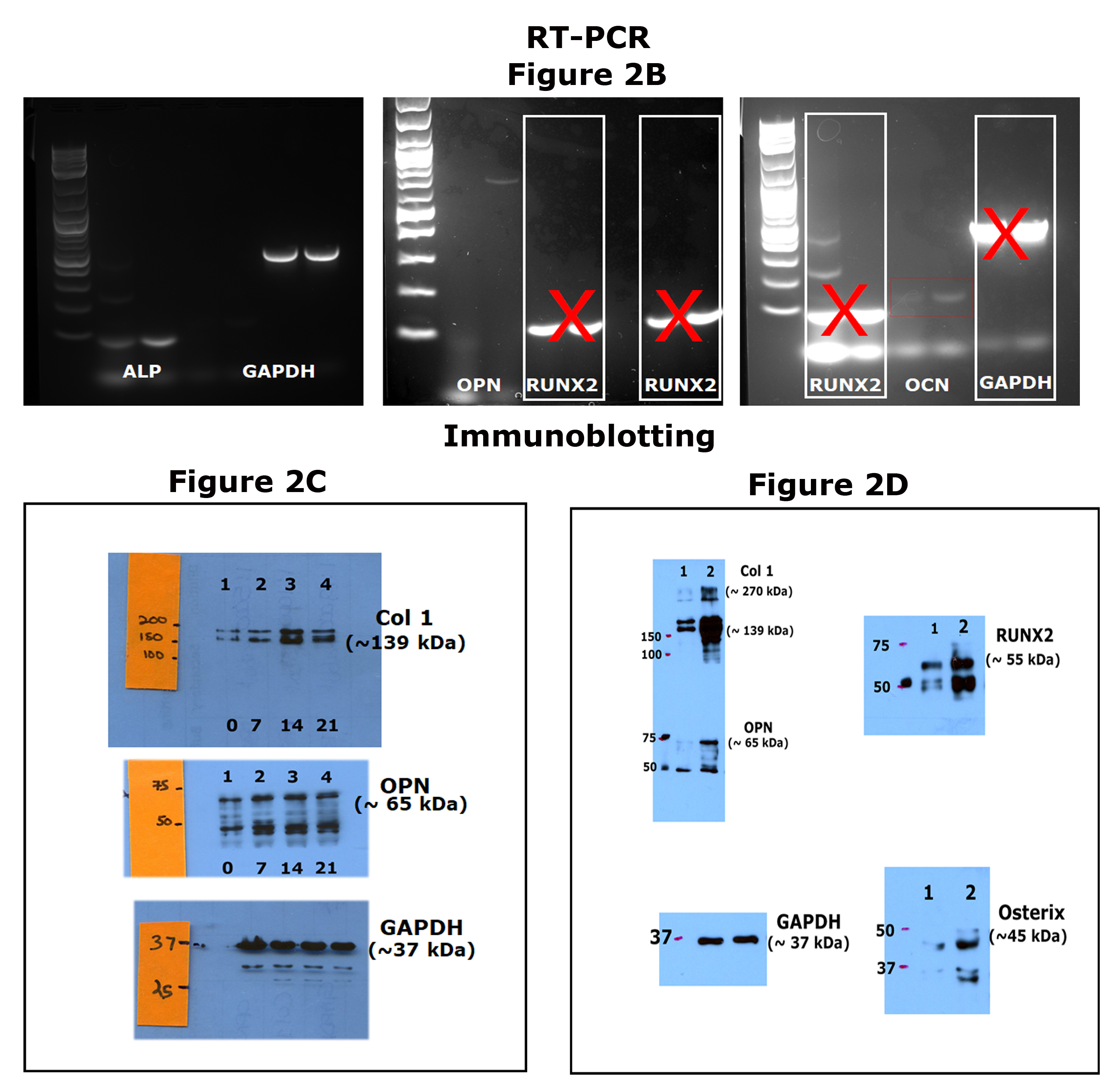

Supplement: S2 Fig — Uncropped raw data provided for 2B (RT-PCR) as well as 2C and D (immunoblotting analysis). (TIF) [file pone.0225598.s002.tif]

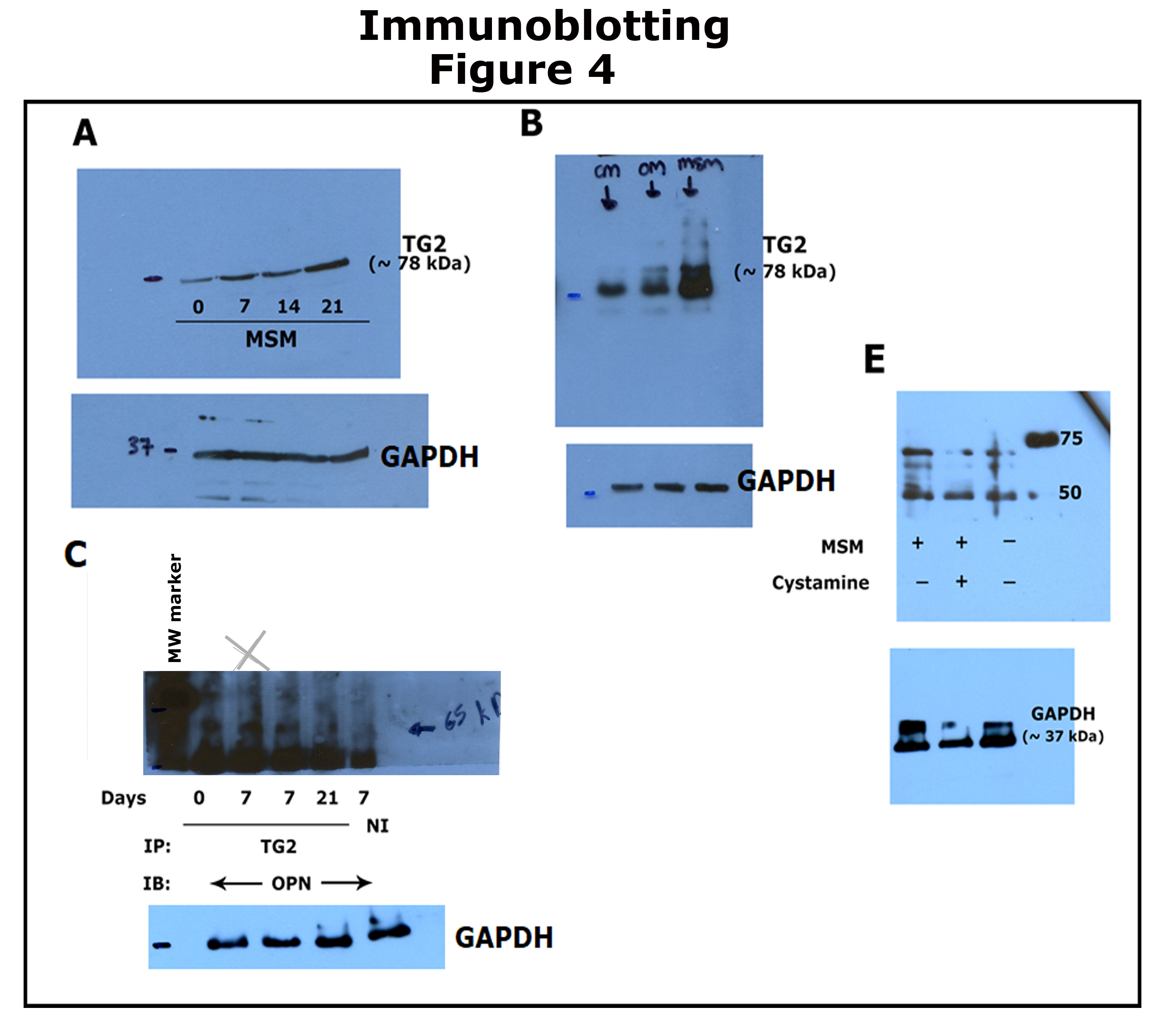

Supplement: S3 Fig — Uncropped raw data provided for 4A-C and E (immunoblotting analysis). (TIF) [file pone.0225598.s003.tif]

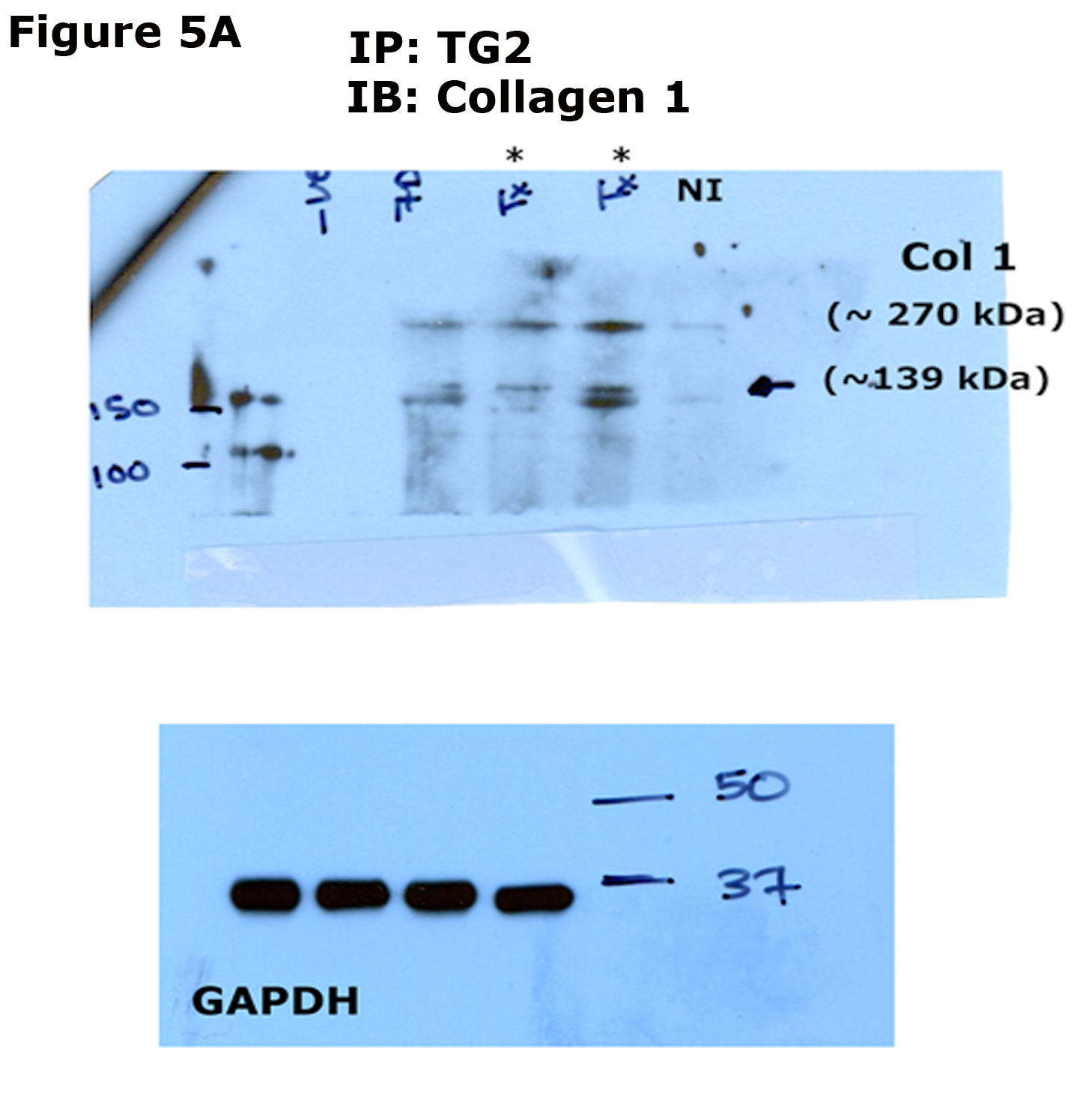

Supplement: S4 Fig — (TIF) [file pone.0225598.s004.tif]
